# Electrophysiological and histological study reveals hidden subclinical haploinsufficiency of *Otof*

**DOI:** 10.1016/j.gendis.2025.101600

**Published:** 2025-03-13

**Authors:** Kyu-Hee Han, Yehree Kim, Kwon Woo Kang, Ju Ang Kim, Yonghee Oh, Min Young Kim, Jin Hee Han, Bong Jik Kim, Eunyoung Yi, Byung Yoon Choi

**Affiliations:** a Department of Otorhinolaryngology, National Medical Center, Seoul 04564, South Korea; b Department of Otorhinolaryngology, Seoul National University Bundang Hospital, Seongnam 13620, South Korea; c College of Pharmacy and Natural Medicine Research Institute, Mokpo National University, Muan 58554, South Korea; d Department of Otolaryngology, HNS and Communicative Disorders, School of Medicine, University of Louisville, Louisville, KY 40202, USA; e Chungnam National University College of Medicine, Chungnam National University Sejong Hospital, Sejong 30099, South Korea

The *OTOF* gene (GenBank AF183185.1) encodes otoferlin, a protein essential for vesicle fusion, synaptic exocytosis, and vesicle replenishment at cochlear inner hair cell synapses, where it functions as a calcium sensor.^1^ A deficiency in otoferlin results in impaired synaptic neurotransmitter release and vesicle recycling,^1^^,^^2^ accompanied by a significant reduction in inner hair cell synapse counts, ultimately leading to prelingual auditory neuropathy spectrum disorder (DFNB9: OMIM 60381).^3^

Notably, otoferlin deficiency does not disrupt cochlear development or the maintenance of cochlear hair cell integrity until well after birth in both humans and mice, rendering these models particularly amenable to gene therapy interventions. Recent studies have explored the potential of gene therapy to restore hearing in individuals with autosomal recessive deafness 9 (DFNB9).^3^ However, despite the successful recovery of hearing thresholds in mouse models following gene therapy, the protein levels of otoferlin remained below 50% of endogenous levels, and there was no significant recovery in the synapse count of inner hair cells (IHCs).^4^^,^^5^ This outcome mirrors the condition of hidden hearing loss in humans, raising the possibility that even successful gene therapy for DFNB9 may only partially mitigate the underlying auditory deficits, with residual hidden hearing loss remaining a likely outcome. Consequently, this study aimed to investigate the histological, audiological, and electrophysiological phenotypes of single heterozygous mice (*Otof*^*+/p.R1934Q*^) carrying the founder allele of DFNB9 found in East Asians (p.R1939Q of *OTOF*), which, while exhibiting normal hearing thresholds, is expected to present reduced otoferlin protein levels compared with wild-type counterparts. We hypothesized that although DFNB9 is an autosomal recessive disorder, normal-hearing single heterozygous carriers of the *OTOF* gene may have hidden audiological defects, such as potential synapse abnormalities, due to reduced protein levels.

The *OTOF* founder variant p.R1939Q of *OTOF* among East Asians corresponds to p.R1934Q in the mouse *Otof* gene (GenBank accession number: NM_001100395.1; Ensembl: ENSMUSG00000062372). Therefore, we generated a C57BL/6 mouse model carrying the common missense variant p.R1934Q at the *Otof* locus on the mouse chromosome using CRISPR/Cas-mediated genome engineering. While *Otof*^*+/+*^ and *Otof*^*+/p.R1934Q*^ mice exhibited normal thresholds, *Otof*^*p.R1934Q/p.R1934Q*^ mice showed no auditory brainstem responses (ABR) (Fig. S1). The distortion product otoacoustic emission responses in both *Otof*^*p.R1934Q/p.R1934Q*^ and *Otof*^*+/p.R1934Q*^ mice were not significantly different from wild-type mice, except at 16 kHz for *Otof*^*p.R1934Q/p.R1934Q*^, and were above noise levels at all frequencies, replicating the auditory neuropathy spectrum disorder phenotype of DFNB9 (Fig. 1A). ABR thresholds did not differ significantly between *Otof*^*+/p.R1934Q*^ and *Otof*^*+/+*^ mice at 2 months for both tone-burst and click stimuli, consistent with DFNB9 inheritance (Fig. 1B). However, ABR wave I amplitudes were significantly reduced in *Otof*^*+/p.R1934Q*^ mice compared with *Otof*^*+/+*^ controls at 4 kHz, 8 kHz, and 16 kHz, and wave II amplitudes also showed a notable reduction at 16 kHz, with wave I showing a more pronounced reduction across frequencies (Fig. 1C, D). This suggests a decrease in fully functional IHCs or synaptic transmission deficits despite normal hearing thresholds in *Otof*^*+/p.R1934Q*^ mice, indicative of hidden hearing loss.

**Figure 1:**
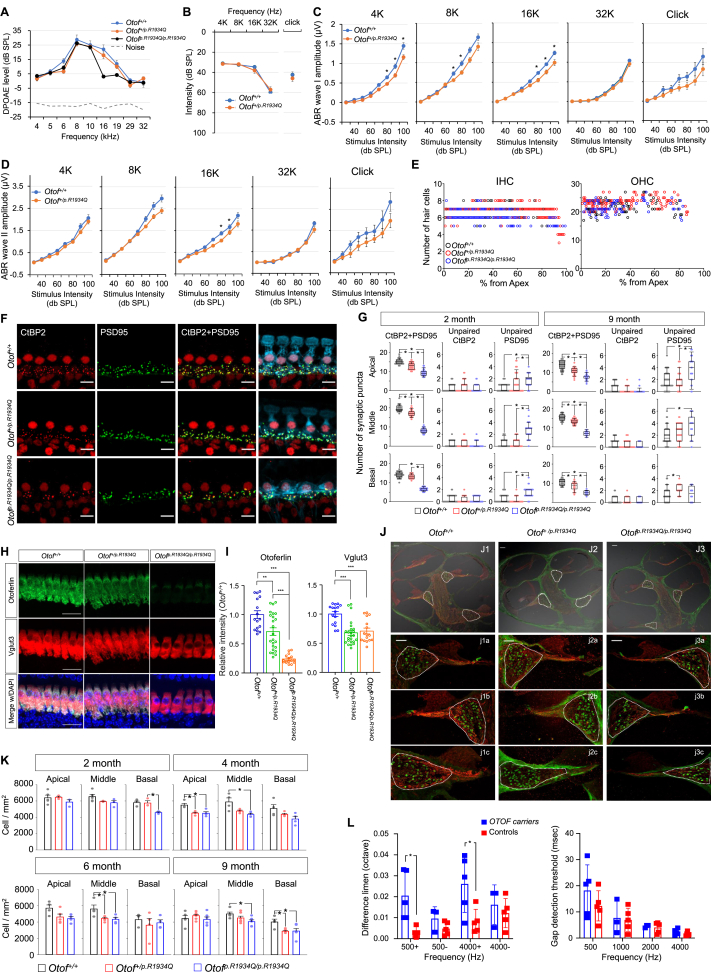
Results of electrophysiological, histological, and psychoacoustic studies related to various *OTOF/Otof* genotypes. **(A)** The distortion product otoacoustic emission (DPOAE) responses in *Otof*^*p.R1934Q/p.R1934Q*^ mice were similar to those of *Otof*^*+/+*^ and *Otof*^*+/p.R1934Q*^ mice, suggesting that *Otof*^*p.R1934Q/p.R1934Q*^ mice exhibit a phenotype consistent with auditory neuropathy spectrum disorder, characterized by a lack of significant auditory brainstem responses (ABR) at 2 months of age (*Otof*^*+/+*^, *n* = 12; *Otof*^*+/p.R1934Q*^, *n* = 22; *Otof*^*p.R1934Q/p.R1934Q*^, *n* = 18). **(B)** The *Otof*^*+/+*^ and *Otof*^*+/p.R1934Q*^ mice equally showed the same normal ABR thresholds for the tone burst and click stimuli for ABR. **(C)** The wave I amplitude of *Otof*^*+/p.R1934Q*^ was significantly lower than that of *Otof*^*+/+*^. This suggests that *Otof*^*+/p.R1934Q*^ mice manifest hidden hearing loss at 2 months of age. **(D)** The amplitude of subsequent wave II was also reduced across the frequencies in *Otof*^*+/p.R1934Q*^ compared with *Otof*^*+/+*^, with a significant reduction at 16 kHz (*Otof*^*+/+*^, *n* = 35; *Otof*^*+/p.R1934Q*^, *n* = 43 for tone burst; *Otof*^*+/+*^, *n* = 14; *Otof*^*+/p.R1934Q*^, *n* = 14 for click). ∗*P* < 0.00625 (student's *t*-test followed by Bonferroni correction). **(E)** The number of hair cells in a segment spanning 1% of the total cochlear length was analyzed. Across all cochlear regions, no significant difference in the number of inner hair cells (IHCs) and outer hair cells (OHCs) is shown among *Otof*^*+/+*^, *Otof*^*+/p.R1934Q*^, and *Otof*^*p.R1934Q/p.R1934Q*^ mice (*n* = 4 for each group). **(F)** Inner hair cell ribbon synapses were observed in *Otof*^*+/+*^, *Otof*^*+/p.R1934Q*^, and *Otof*^*p.R1934Q/p.R1934Q*^ mice. Representative images of inner hair cells were immunolabeled with anti-CtBP2 (red), anti-PSD95 (green), and anti-calretinin (cyan). These images were acquired from the middle turns of 6-month-old cochleae from *Otof*^*+/+*^, *Otof*^*+/p.R1934Q*^, and *Otof*^*p.R1934Q/p.R1934Q*^ mice. Scale bar: 10 μm. **(G)** Profile plots of the number of paired and unpaired CtBP2 and PSD95 puncta. The number of cells and cochleae used for quantification were as follows: 2-month-old cochleae (*Otof*^*+/+*^, 41–57 IHCs from *n* = 4; *Otof*^*+/p.R1934Q*^, 36–54 IHCs from *n* = 4 or 5; *Otof*^*p.R1934Q/p.R1934Q*^, 30–42 IHCs from *n* = 5), 9-month-old cochleae (*Otof*^*+/+*^, 38–63 IHCs from *n* = 4 or 5; *Otof*^*+/p.R1934Q*^, 23–49 IHCs from *n* = 3–6; *Otof*^*p.R1934Q/p.R1934Q*^, 16–23 IHCs from *n* = 3 or 4). The solid line in the box indicates the median. The box and whiskers indicate interquartile and 10–90 percentile range. ∗*P* < 0.05 (ANOVA on Rank followed by Dunn's test). **(H, I)** Z-projected confocal images of cochlear IHCs of indicated genotypes of *Otof*^*+/+*^, *Otof*^*+/p.R1934Q*^, and *Otof*^*p.R1934Q/p.R1934Q*^ mice were analyzed for comparison of normalized fluorescence intensity of otoferlin and Vglut3 (*Otof*^*+/+*^, 16 IHCs from *n* = 4; *Otof*^*+/p.R1934Q*^, 24 IHCs from *n* = 6; and *Otof*^*p.R1934Q/p.R1934Q*^, 16 IHCs from *n* = 4). Otoferlin immunofluorescence levels decreased in *Otof*^*+/p.R1934Q*^ mice to 71% and in *Otof*^*p.R1934Q/p.R1934Q*^ mice to 24% as compared with *Otof*^+/+^ controls. Vglut3 immunofluorescence also exhibited a decrease in both *Otof*^*+/p.R1934Q*^ and *Otof*^*p.R1934Q/p.R1934*^ mice, with comparable decreases of 68% and 71% of *Otof*^+/+^ controls respectively. Scale bar: 20 μm ∗∗*P* < 0.01 and ∗∗∗*P* < 0.001 (one-way ANOVA followed by Tukey test). **(J)** Spiral ganglion neuron (SGN) density of *Otof*^*+/+*^, *Otof*^*+/p.R1934Q*^, and *Otof*^*p.R1934Q/p.R1934Q*^ mice. (J1–3) Mid-modiolar sections of the cochlear were immunolabeled with anti-calretinin (green) and anti-neurofilament (red). These images were acquired from 4-month-old *Otof*^*+/+*^, *Otof*^*+/p.R1934Q*^, and *Otof*^*p.R1934Q/p.R1934Q*^ mice. Scale bar: 100 μm. (j1–3) Enlarged views of the spiral ganglia corresponding to the apical (j1a, j2a, j3a), middle (j1b, j2b, j3b), and basal (j1c, j2c, j3c) turns of cochlea. Scale bar: 50 μm. **(K)** Profile plot of the SGN density in 2-, 4-, 6-, and 9-month-old *Otof*^*+/+*^ (3–5 cochleae), *Otof*^*+/p.R1934Q*^ (3–7 cochleae), and *Otof*^*p.R1934Q/p.R1934Q*^ (3–6 cochleae) mice. Data are presented as mean ± standard error of the mean. ∗*P* < 0.05 (one-way ANOVA followed by Tukey test). **(L)** Results of frequency discrimination limen test (left panel) and gap detection test (right panel) from human *OTOF* carriers and their age-matched controls. In the left panel, “+” denotes when the task is to differentiate sounds that are higher in pitch than the reference sound, and “–” denotes when the task is to differentiate sounds that are lower in pitch than the reference sound. *n* = 5 for both the *OTOF* carriers and their age-matched controls. *OTOF* carriers carried the variants p.Arg1939Gln (*n* = 2), p.Arg1856Trp (*n* = 2), and p.Arg1735Glyfs∗28. ∗*P* < 0.02 (Mann–Whitney *U* test).

To explore whether the decreased wave I amplitude in *Otof*^*+/p.R1934Q*^ reflects a decrease in the number of fully functional IHCs rather than deficits in synaptic transmission and whether the significant deficiency of functional otoferlin causes cochlear hair cell loss, IHC and outer hair cell (OHC) numbers were compared among *Otof*^*+/+*^, *Otof*^*+/p.R1934Q*^, and *Otof*^*p.R1934Q/p.R1934Q*^ mice, showing no significant differences (Fig. S2; Fig. 1E). This confirmed that the hearing deficit in *Otof*^*+/p.R1934Q*^ and *Otof*^*p.R1934Q/p.R1934Q*^ mice would not be directly due to IHC loss.

Therefore, a deficit in synaptic transmission was suspected as the cause of decreased wave I, a subsequent decrease in wave II amplitude in *Otof*^*+/p.R1934Q*^, and no ABR response from *Otof*^*p.R1934Q/p.R1934Q*^ mice. To prove this, IHC ribbon synapses were examined across genotypes. Cochleae from 2-, 4-, 6-, and 9-month-old *Otof*^*+/+*^, *Otof*^*+/p.R1934Q*^, and *Otof*^*p.R1934Q/p.R1934Q*^ mice were immunolabeled for presynaptic ribbons (CtBP2) and postsynaptic components (PSD95 or GluA2). Most CtBP2 puncta were juxtaposed to PSD95 or GluA2 puncta, with few unpaired. First, examination of IHC ribbon synapses across genotypes revealed significantly fewer paired synaptic puncta (CtBP2+PSD95 and CtBP2+GluA2) in *Otof*^*p.R1934Q/p.R1934Q*^ mice, correlating with severe hearing deficits (Fig. 1F, G; Fig. S3, 4). Notably and unexpectedly, *Otof*^*+/p.R1934Q*^ mice had slightly fewer paired synapses than *Otof*^*+/+*^ mice, with significant reductions observed from 2 to 9 months more prominent in the apical and middle cochlear turn. The pattern of paired synaptic puncta reduction was similar across age groups, with CtBP2+PSD95 differences appearing earlier (even at 2 months of age) than those involving CtBP2+GluA2 beginning at 4 months. This pronounced reduction in apical and middle turns aligned with ABR wave I amplitude reductions at low to mid frequencies, further supporting the hidden hearing loss phenotype.

Given no significant differences in IHC and OHC numbers, we examined *Otof* mRNA and protein levels across genotypes. While *Otof* mRNA levels remained similar, protein levels were slightly reduced in *Otof*^*+/p.R1934Q*^ mice, and this reduction was more pronounced in *Otof*^*p.R1934Q/p.R1934Q*^ mice (Fig. H, I; Fig. S5). Therefore, the reduced otoferlin level may affect the observed audiological phenotype in *Otof*^*+/p.R1934Q*^ and *Otof*^*p.R1934Q/p.R1934Q*^ mice.

Finally, spiral ganglion neuron density (SGN) was compared among genotypes. Significant SGN loss was observed in the basal turn of 2-month-old *Otof*^*p.R1934Q/p.R1934Q*^ mice, with no significant differences in other cochlear turns (Fig. 1J, K). *Otof*^*+/p.R1934Q*^ mice first showed lower SGN density compared with *Otof*^*+/+*^ at 4 months. These findings indicate that synaptic disruption indicated by fewer paired CtBP2 and PSD95 puncta starting as early as 2 months of age precedes SGN loss in the *Otof*^*+/p.R1934Q*^ mice.

Furthermore, we sought to expand this research into a human pilot study to examine whether similar findings in rodents could be observed in normal-hearing individuals who are single heterozygous carriers of the *OTOF* variant. ABR wave I is difficult to measure in humans, therefore we utilized the frequency discrimination limen test and gap detection test commonly used to assess auditory neuropathy spectrum disorder. Despite normal hearing thresholds, these carriers struggled with frequency discrimination tasks, particularly at 500 and 4000 Hz (Fig. 1L, left panel). Gap detection thresholds were also slightly poorer at lower frequencies (Fig. 1L, right panel). Although the number of participants is small, these findings suggest subclinical deficits in the carriers aligning with the decreased ABR wave I amplitude observed in *Otof*^*+/p.R1934Q*^ mice, particularly at low and mid frequencies.

The findings of this study reveal that single heterozygous carriers of the *Otof* mutant allele, previously presumed to have a normal phenotype, in fact, exhibit a subtle yet discernible audiological phenotype consistent with hidden hearing loss through a haploinsufficiency mechanism. Consequently, the parents or normal-hearing, single heterozygous carrier siblings of DFNB9 patients may themselves possess hidden hearing loss, indicating a potentially increased vulnerability to external factors such as noise. Furthermore, these results suggest that even patients who undergo successful *OTOF* gene therapy, with a restoration of hearing thresholds to normal levels, may still harbor various subclinical audiological deficits, including hidden hearing loss. This underscores the need for further research to explore the full spectrum of the auditory phenotype of single heterozygous carriers of *OTOF* in the general population as well as DFNB9 patients and their immediate families.

## CRediT authorship contribution statement

**Kyu-Hee Han:** Writing – original draft, Visualization, Investigation, Formal analysis. **Yehree Kim:** Writing – original draft, Visualization, Investigation, Formal analysis. **Kwon Woo Kang:** Writing – original draft, Visualization, Investigation, Formal analysis. **Ju Ang Kim:** Writing – original draft, Visualization, Formal analysis. **Yonghee Oh:** Methodology. **Min Young Kim:** Visualization, Investigation, Formal analysis. **Jin Hee Han:** Formal analysis, Data curation. **Bong Jik Kim:** Writing – review & editing. **Eunyoung Yi:** Writing – review & editing, Supervision, Project administration, Funding acquisition, Conceptualization. **Byung Yoon Choi:** Writing – review & editing, Supervision, Project administration, Funding acquisition, Conceptualization.

## Ethics declaration

The human study was approved by the Institutional Review Board of Seoul National University Bundang Hospital (IRB No. B-1007-105-402). The animal study was approved by the Institutional Animal Care and Use Committee of Seoul National University Bundang Hospital (BA-2210-353-001) and Mokpo National University (MNU-IACUC-2022-009, MNU-IACUC-2023-003).

## Funding

This study is supported by the Basic Science Research Program through the National Research Foundation (NRF), funded by the Ministry of Education of South Korea (No. 2021R1A2C2092038 to Choi. B.Y.; 2022R1I1A3072036 to Yi, E.), in part by Glocal University Project of Mokpo National University in 2024 (to Yi, E.), and the Bio Core Facility center program through the NRF (South Korea) (No. 2022M3A9G1014007 to Choi, B.Y.). Additional funding was provided by the Basic Research Laboratory Program through the NRF, funded by the Ministry of Education of South Korea (No. RS-2023-0021971031482092640001 to Choi, B.Y.) and the Technology Innovation Program (K_G012002572001 to Choi. B.Y.) funded By the Ministry of Trade, Industry and Energy. This study is also funded by SNUBH intramural research fund (South Korea) (No. 13-2022-0010, 02-2017-0060, 16-2023-0002, 13-2023-0002,16-2022-0005, 13-2024-0004, and 13-2017-0013 to Choi. B.Y.) and the SNUBH-Basic Co-Research Fund (South Korea) (No. 16-2024-0011).

## Conflict of interests

The authors declared no conflict of interests.

## References

[1] Roux I., Safieddine S., Nouvian R. (2006). Otoferlin, defective in a human deafness form, is essential for exocytosis at the auditory ribbon synapse. Cell.

[2] Strenzke N., Chakrabarti R., Al-Moyed H. (2016). Hair cell synaptic dysfunction, auditory fatigue and thermal sensitivity in otoferlin Ile515Thr mutants. EMBO J.

[3] Rodríguez-Ballesteros M., del Castillo F.J., Martín Y. (2003). Auditory neuropathy in patients carrying mutations in the otoferlin gene (*OTOF*). Hum Mutat.

[4] Lv J., Wang H., Cheng X. (2024). AAV1-hOTOF gene therapy for autosomal recessive deafness 9: a single-arm trial. Lancet.

[5] Al-Moyed H., Cepeda A.P., Jung S., Moser T., Kügler S., Reisinger E. (2019). A dual-AAV approach restores fast exocytosis and partially rescues auditory function in deaf otoferlin knock-out mice. EMBO Mol Med.

