# Supplementary material for: Electrophysiological and histological study reveals hidden subclinical haploinsufficiency of Otof
Source: Genes Dis. 2025 Mar 13;13(2):101600. doi: 10.1016/j.gendis.2025.101600 (PMC12596599; doi:10.1016/j.gendis.2025.101600)
Supplement: Multimedia component 1 [file mmc1.docx]

**Materials and Methods**

**Generation of a mouse *Otof^p.R1934Q/p.R1934Q^* model**

A C57BL/6 mouse model with a prevalent missense p.R1934Q variant at the orthologous mouse *Otof* locus was created using CRISPR/Cas-mediated genome engineering. This variant corresponds to the founder variant of *OTOF* p.R1939Q among East Asians in the mouse *Otof* gene (GenBank accession number: NM_001100395.1; Ensembl: ENSMUSG00000062372). Forty-seven exons have been identified for mouse *Otof*, with the ATG start codon in exon 1 and TGA stop codon in exon 47. Codon 1934, located on exon 47, was selected as the target site. A guide RNA (gRNA) targeting vector and donor oligo (with the targeting sequence, flanked by 120 bp homologous sequences combined on both sides) were designed as follows: gRNA1 (matches forward strand of gene): 5'CCACCCTAGCCGGCCTGACACGG; gRNA2 (matches reverse strand of gene): 5'ATGCCGTGTCAGGCCGGCTAGGG. The p.R1934Q (CGG to CAG) variant site in the donor oligo was introduced into exon 47 by homology-directed repair. A silent mutation (ACG to ACC) was also introduced to prevent the binding and re-cutting of the sequence by gRNA after homology-directed repair. The donor oligo sequence was 5'TGTATGGGTGGATGGTGCTGGTTATCTGACCAGCCCCTCCTGTCTTCTCCCCACCCTAGCCAGCCTGACACCGCATTCGTCTGGTTCCTGAACCCACTCAAATCTATCAAGTACCTCATCTGC. The target region of mouse *Otof* locus was amplified by polymerase chain reaction (PCR) with specific primers. For *Otof^+/p.R1934Q^* knock-in mouse production, Cas9 messenger RNA (mRNA), a gRNA generated by in vitro transcription, and donor oligo were co-injected into fertilized eggs. The F0 pups were genotyped by PCR amplification using the following primers (mouse *Otof*-F: 5'ACCAGGGTCTGCCTCAATTCATC; mouse *Otof*-R: 5'ATGAAGTCCAGCAAGCAGGTGTCT). This was followed by sequence analysis to confirm that the p.R1934Q (CGG to CAG) variant was successfully introduced.

**Auditory Measurements**

ABRs and DPOAEs were measured with SmartEP (Intelligent Hearing Systems, Miami, FL, USA) in an electrically and acoustically shielded box as previously described^1,2^. Mice were sedated with intraperitoneal injection of a mixture of zolazepam/tiletamine (50 mg/kg, Zoletil 50; Virbac, Carros, France) and 10 mg/kg xylazine (Rompun; Bayer Health Care, Leverkusen, Germany). ABR signals were measured from a subdermal needle electrode inserted at right mastoid area referenced to an electrode placed at the vertex, and a ground electrode was inserted at contralateral mastoid. ABRs were recorded using high-frequency software (version 2.33) and high-frequency transducers (HFT9911-20-0035, IHS, Miami, FL, USA). Click stimuli (31 µs duration) were presented through ER3 Insert Earphones with a rectangular envelope, while 4, 8, 16, and 32 kHz tone bursts (1,562 µs duration) were delivered via high-frequency transducers gated with a Blackman envelope (2.5 ms rise/fall time). Stimuli were presented from 100 dB SPL to 0 dB SPL with 10 dB decrements. Evoked potentials were amplified, filtered, and averaged across 512 sweeps. The threshold was considered as the minimal stimulus intensity capable of eliciting a distinguishable response, as evaluated by two investigators. The amplitude of wave I and II was manually assessed by measuring from the baseline to the peak of the first and the second positive depletion in the ABR waveform. DPOAEs were measured to assess the function of cochlear outer hair cells. Using SmartDPOAE (version 5.33, IHS), DPOAEs were recorded for F2 ranging from 4 to 32 kHz. An Etymotic 10B+ OAE microphone probe tip was positioned in the external auditory canal alongside an IHS high frequency transducer, delivering auditory stimuli composed of two pure tones with distinct frequencies (F2 = 1.22 × F1, L1=65 dB, L2=55 dB). A total of eight blocks were acquired, with each block consisting of 16 sweeps.

**Immunohistochemistry of the cochlear tissue and quantification of protein**

Mice of either sex at indicated age were euthanized (sevoflurane inhalation followed by decapitation) and cochleae were collected. The cochleae were perfused with 4% ice-cold paraformaldehyde through oval or round window and post-fixed at 4 ℃ for 1 hour. Cochlear turns were excised by delicately removing bony capsule, lateral wall, and tectorial membrane for immunohistochemistry. Tissues were incubated in a blocking/permeabilizing buffer composed of PBS with 5% goat serum and 0.25% Triton X-100. Then samples were incubated with primary polyclonal antibodies: anti-Otoferlin (1:100; mouse monoclonal, ab53233, Abcam) and anti-Vglut3 (1:200; rabbit polyclonal, 135203, Synaptic systems) antibodies, in blocking solution overnight at 4℃. After PBS washes, cochlear turns were incubated with fluorescent secondary antibodies (1:400, goat anti-mouse, A11017, Invitrogen; 1:400, 555 goat anti-rabbit, A21428, Molecular probe) in blocking/permeabilizing solution overnight at room temperature. After washing with PBS, cochlear tissues were mounted with DAPI on slide glasses and coverslipped.

Samples were imaged using a confocal laser scanning microscope (LSM800, Zeiss). The intracellular fluorescence of the protein was assessed using the *ImageJ* program. The quantification was performed by calculating the Corrected Total Cell Fluorescence (CTCF) for each fluorescent channel as previously described^3, 4^. Briefly, the contour of the IHC was selected on sum projections of z-stack images and the integrated density and area were measured. Background fluorescence was quantified by measuring mean intensity in a fluorescence-free region adjacent to the IHC and subtracted using formula: CTCF=Integrated Density - (Area of ROI - Mean Background Intensity).

**Hair cell count**

Fixed cochlea was decalcified by immersion in 5% ethylenediamine tetracetate (EDTA) at 4℃ for 24 hours. The decalcified otic capsule, stria vascularis, tectorial membrane and Reissner’s membrane were removed. The remaining tissue containing the organ of Corti was subjected to immunofluorescence-labeling procedures as previously described^5^. Briefly, the tissue was immersed in blocking/permeabilization buffer (phosphate buffered saline supplemented with 5% normal goat serum and 0.25 % Triton X-100, pH 7.4) for 1 hour at room temperature. Hair cells were then immunofluorescence-labeled with rabbit polyclonal anti-Myosin 6 (1:500, overnight, 4 ℃, M5187, Sigma Aldrich) and goat anti-rabbit Alexa Fluor 555 (1:1000, 1 hour, room temperature, A21428, Invitrogen). The labeled tissue was mounted on a slide using FluorSave anti-fade reagent. Myosin 6-positive hair cells were imaged using laser scanning confocal microscope (LSM710, Zeiss) and the composite image of the entire length of the organ of Corti was reconstructed in ImageJ. The number of IHCs and OHCs in each segment spanning 1% of the total length were manually quantified. The segments that had been damaged during tissue preparation were identified under DIC optics and excluded from hair cell counting analysis.

**Ribbon synapse quantification**

The region containing the organ of Corti was carefully dissected out from fixed cochlea and was subjected to immunofluorescence-labeling procedures as described above. After blocking/permeabilization (phosphate buffered saline containing 5% normal goat or donkey serum and 0.25% Triton X-100, pH 7.4) the cochlear tissue was reacted with primary antibodies overnight. The presynaptic ribbon protein CtBP2 was detected with rabbit polyclonal anti-CtBP2 (1:500, BS2287, Bioworld). The postsynaptic components at the cochlear afferent fiber terminals were labeled with either mouse monoclonal anti-GluA2 (1:500, MAB397, Merck) or mouse monoclonal anti-PSD-95 (1:250, 75-028, Neuromab). Additionally, IHCs and a subset of cochlear afferent fibers were co-labeled with goat polyclonal anti-calretinin (1:500, CG1, Swant). The secondary antibodies were used as a combination of either goat anti-mouse Alexa Fluor 488 (A32723) and goat anti-rabbit Alexa Fluor 555(A21428) or donkey anti-mouse Alexa Fluor 488 (A21206), donkey anti-rabbit Alexa Fluor 555 (A31572) and donkey anti-goat Alexa Fluor 633 (A21082). All secondary antibodies were from Invitrogen and were used at 1:1000. The high-resolution confocal z-stack images were obtained using a confocal laser scanning microscope (LSM 710, Zeiss). The pre- (CtBP2) and postsynaptic (GluA2 or PSD-95) puncta within 0.5 µm from each other were considered ‘paired’.

**Spiral Ganglion Neuron (SGN) density**

Fixed and decalcified cochleae were cryoprotected by sequential immersion in mixtures of 30% sucrose and OCT compound (100:0, 75:25, 50:50, 25:75, 0:100) at 4℃ over 18-hour period and were frozen. The cochlear tissue was cryosectioned at 10 µm thickness (Leica, CM1860), mounted on Superfrost microscope slides^6^ and stored at -20℃ until use. To immunolabel the spiral ganglion neurons the sectioned cochlear tissue was rinsed twice with PBS, treated with blocking/permeabilizing buffer for 1 hour, and then incubated with primary antibodies at 4℃ overnight. The primary antibodies used were mouse monoclonal anti-calretinin (1:500, MAB1568, Merck) and chicken polyclonal anti-neurofilament heavy (1:1000, ab4680, Abcam). Next day, the cochlear tissue was rinsed three times with blocking buffer and incubated with goat anti-mouse Alexa Flour 488 (1:1000, A32723) and goat anti-chicken Alexa Flour 633 (1:1000, A21449, Invitrogen) for 1 hour at room temperature. After 3 rinses the tissue was treated with FluorSave anti-fade reagent and coverslipped. The spiral ganglion areas were imaged using a confocal laser scanning microscope (LSM 710, Zeiss) and the number of neurofilament-positive neurons in each ganglion was counted.

**Real-time PCR**

Total RNA was extracted from the whole cochlea of mice (p60). TRIZOL reagent (15596026, Invitrogen) was used for RNA extraction following manufacturer’s protocol. cDNA synthesis was carried out according to SuperscriptIII First-Strand (18080-51, Invitrogen) protocol. Real-Time PCR was performed using Taqman™ Fast Advanced Master Mix (4444557, Applied Biosystems), Mm00453306_m1 (4331182) for *Otof* and Mm99999915_g1 (4448489) for *Gapdh*. PCR amplification was carried out using QuantStudio™ 7 Flex Real-time PCR system (Applied Biosystems) with cycling conditions comprising an initial step at 50℃ for 2 min and 95℃ for 2 min, followed by 40 cycles of 95℃ for 1 sec and 60℃ for 20 sec.

**RNAscope^®^ *in situ* hybridization**

Both cochleae were extracted, and serial sections were made at 5-μm thickness. The tissue sections were mounted onto the SuperFrost^®^ Plus slides, and air-dried. Sections were fixed in 4% PFA at 4℃ for 15 min. The fixed sections were dehydrated using a sequential ethanol treatment: 50% EtOH for 5 min, 70% EtOH for 5min, and two round of 100% EtOH for 5 min each at room temperature and then air-dried for 5 min. The tissue sections were subjected to RNAscope^®^ (ACDbio, Newark, CA) *in situ* hybridization. RNAscope® Fluorescent Multiplex Assay (ACDbio, Cat. No. 320851) was done according to the manufacturer’s instructions. Briefly, sections were treated with RNAscope® Protease IV for 30 min at room temperature, then hybridized with the RNAscope Probe-Mm-Otof-C1 (ACDbio, Cat. No. 485671) and Probe-Mm-Slc17a8-C2 (ACDbio, Cat. No. 431261-C2) for 2 hours at 40℃. Signal amplification was achieved using series of amplifiers. Tissues were counterstained with DAPI and the fluorescent signals were visualized using a confocal microscope (LSM800, Zeiss)..

**Frequency discrimination limen test and gap detection test**

We recruited five single heterozygous carriers of the *OTOF* variant and their age-matched controls to participate in the study. The carriers carried the variants p.Arg1856Trp (n=2), p.Arg1939Gln (n=2), and p.Arg1735Glyfs*28. The institutional review board of the Seoul National University Bundang Hospital approved this study.

All experiments were conducted in a double-walled, sound attenuated booth. Signals were generated with MATLAB (version R2010b), processed through an ESI Juli sound card, TDT PA5 digital attenuator and HB7 headphone buffer, and presented over Sennheiser HD-25 headphones. Each headphone’s frequency response was equalized using calibration measurements obtained with a Brüel & Kjær sound level meter with a 1-inch microphone in an artificial ear. Prior to all experiments, loudness balancing was conducted for a subset of test frequencies (0.125, 0.175, 0.25, 0.375, 0.5, 0.625, 0.75, 0.875, 1, 2, 3, and 4 kHz) using a method of adjustment. For frequency discrimination limen test, stimuli were presented monoaurally to both ears and a 1-up, 2-down adaptive procedure was used to determine a threshold at the 71% correct response. Tones of 400-ms duration were presented in each of three intervals in a three alternative forced choice adaptive paradigm. The first interval was used as the reference stimulus, and the interval containing the different tone was randomly selected from the second and third intervals. Subjects were instructed to determine whether the second or third interval differed from the first interval. The size of the frequency difference was reduced by a factor s after two consecutive correct responses and was increased by the same factor after an incorrect response. The value of s was initially set at 2, and was reduced to 1.41 after the initial two reversals. Each run continued until there were a total of eight reversals and the threshold was estimated as the mean of the last six. For gap detection test, a two-interval two-alternative forced-choice task was used, where the temporal gap occurring randomly in one of the two intervals of each trial. A run was composed of 54 trials at a fixed gap duration, the responses for the first four trials being discarded. All stimuli were generated with the fixed 5-ms raised-cosine onset/offset ramps. A percent-correct score was calculated and the data points were used to construct psychometric functions for each of the test frequencies in each of the experimental conditions.

**Supplementary Figures**

**
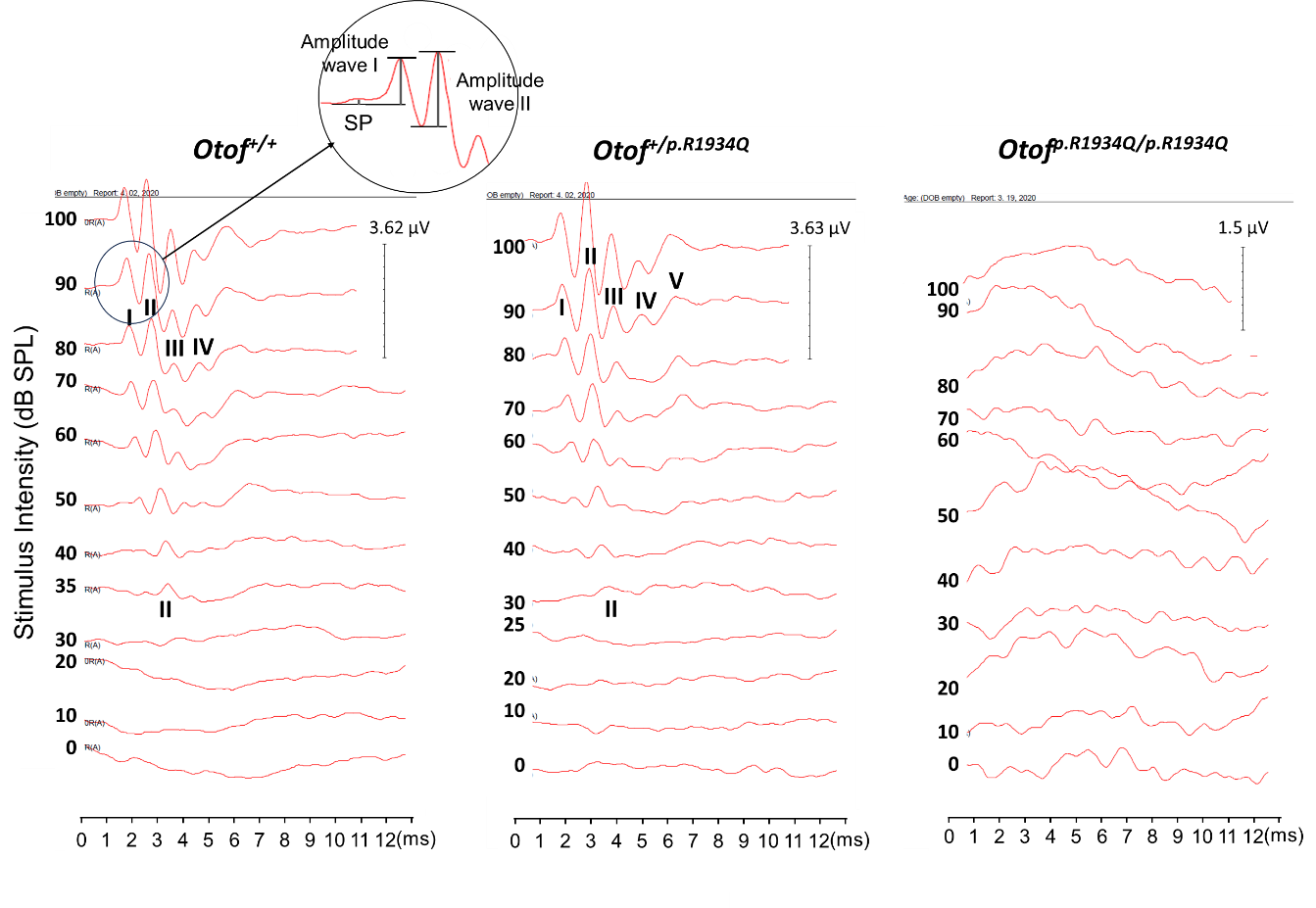
**

**Supplementary Figure 1.** *Otof^p.R1934Q/p.R1934Q^* mice display no significant ABR up to 100dB of stimulus at 16kHz, whereas *Otof^+/+^* and *Otof^+/p.R1934Q^* still maintain normal ABR threshold at age 2 months. (Insert: ABR wave I amplitude was measured from baseline to peak, and wave II from preceding trough to peak. summating potential SP)

**
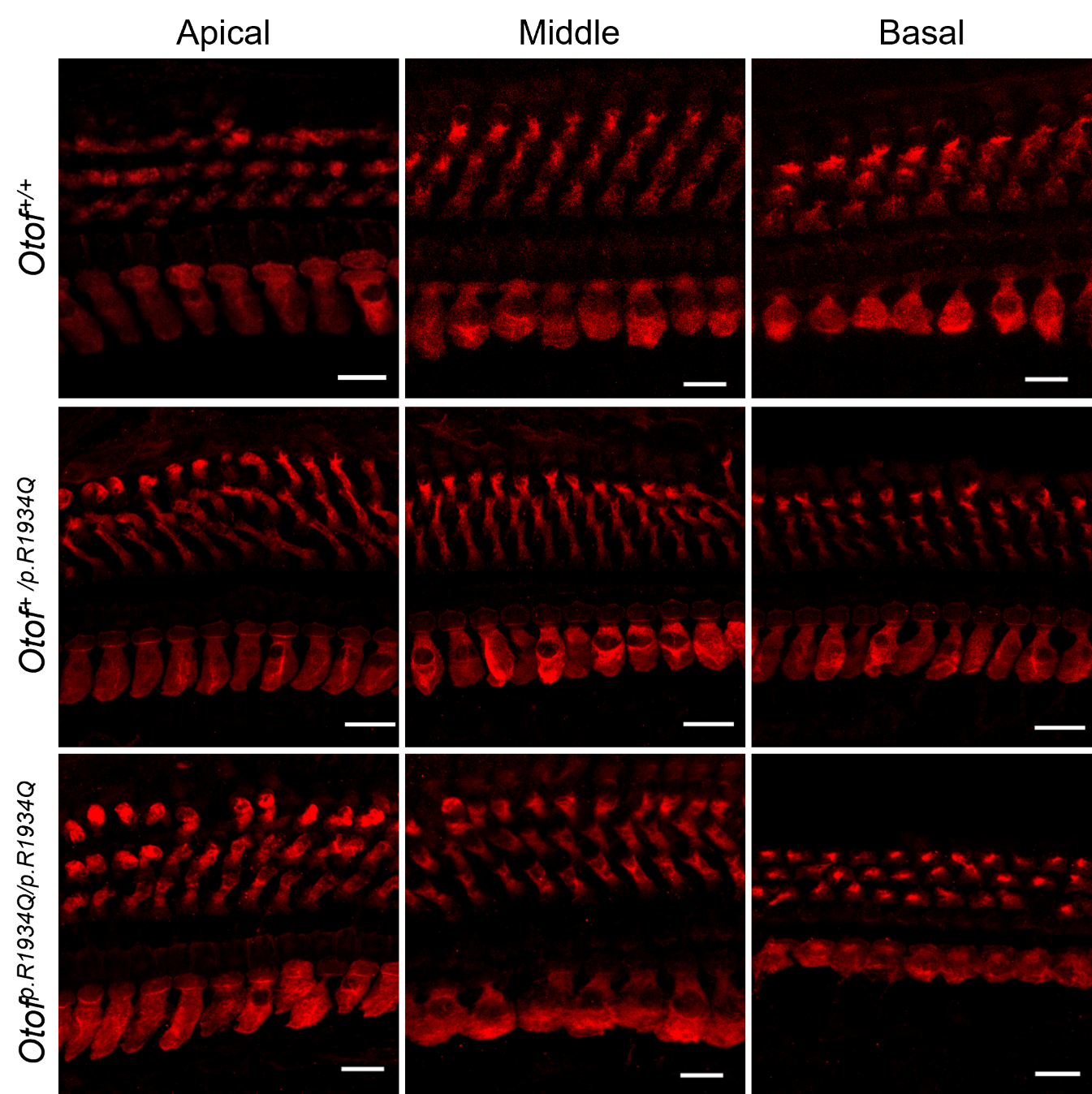
**

**Supplementary Figure 2.** Representative images of hair cells in apical (10-20% from the apex), middle (45-55% from the apex), and basal turns (75-85% from the apex) from *Otof^+/+^*, *Otof^+/p.R1934Q^* and *Otof^p.R1934Q^/^p.R1934Q^ mice*. IHCs and OHCs are immunolabeled with anti-myosin 6 (red). Scale bar: 10 µm.

**
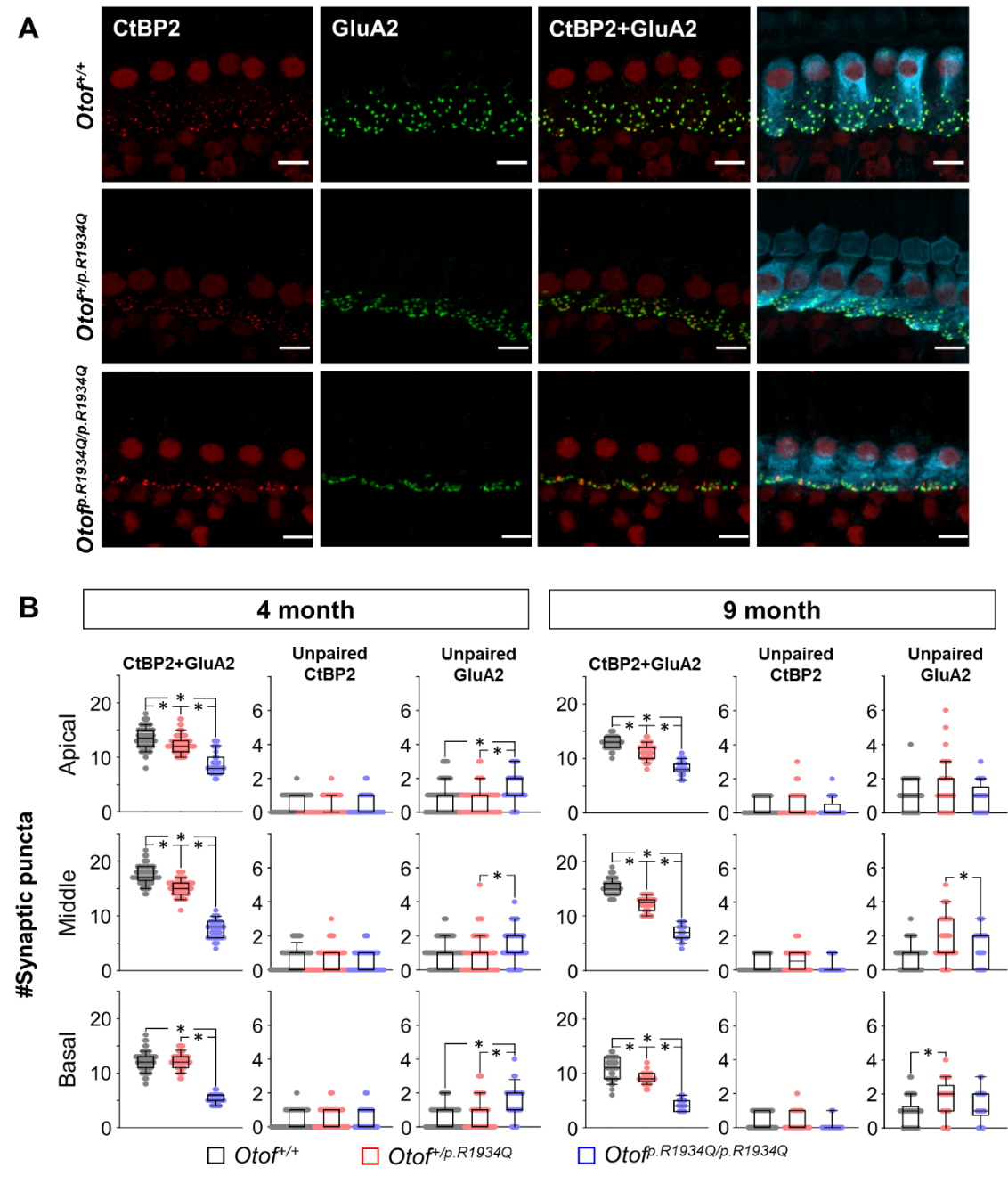
**

**Supplementary figure 3. (A)** Representative images of inner hair cells immunolabeled with anti-CtBP2 (red), anti-GluA2 (green), and anti-calretinin (cyan) were acquired from the middle turns of 2-month-old cochleae from *Otof^+/+^*, *Otof^+/p.R1934Q^*, and *Otof^p.R1934Q/p.R1934Q^* mice. Scale bar: 10 µm. **(B)** Profile plots of the number of paired and unpaired CtBP2 and GluA2 puncta. The number of cells and cochleae used for quantification were as follows: 4-month-old cochleae (*Otof^+/+^*, 60-73 IHC from n=5; *Otof^+/p.R1934Q^*, 52-77 IHC from n=5-6; *Otof^p.R1934Q/p.R1934Q^*, 31-50 IHC from n=5), 9-months-old cochleae (*Otof^+/+^*, 38-49 IHC from n=4; *Otof^+/p.R1934Q^*, 29-42 IHC from n=4; *Otof^p.R1934Q/p.R1934Q^*, 18-25 IHC from n=3-4). Solid line in the box indicates the median. The box and whiskers indicate interquartile and 10-90 percentile range. * p<0.05 (ANOVA on Rank followed by Dunn’s test).

**
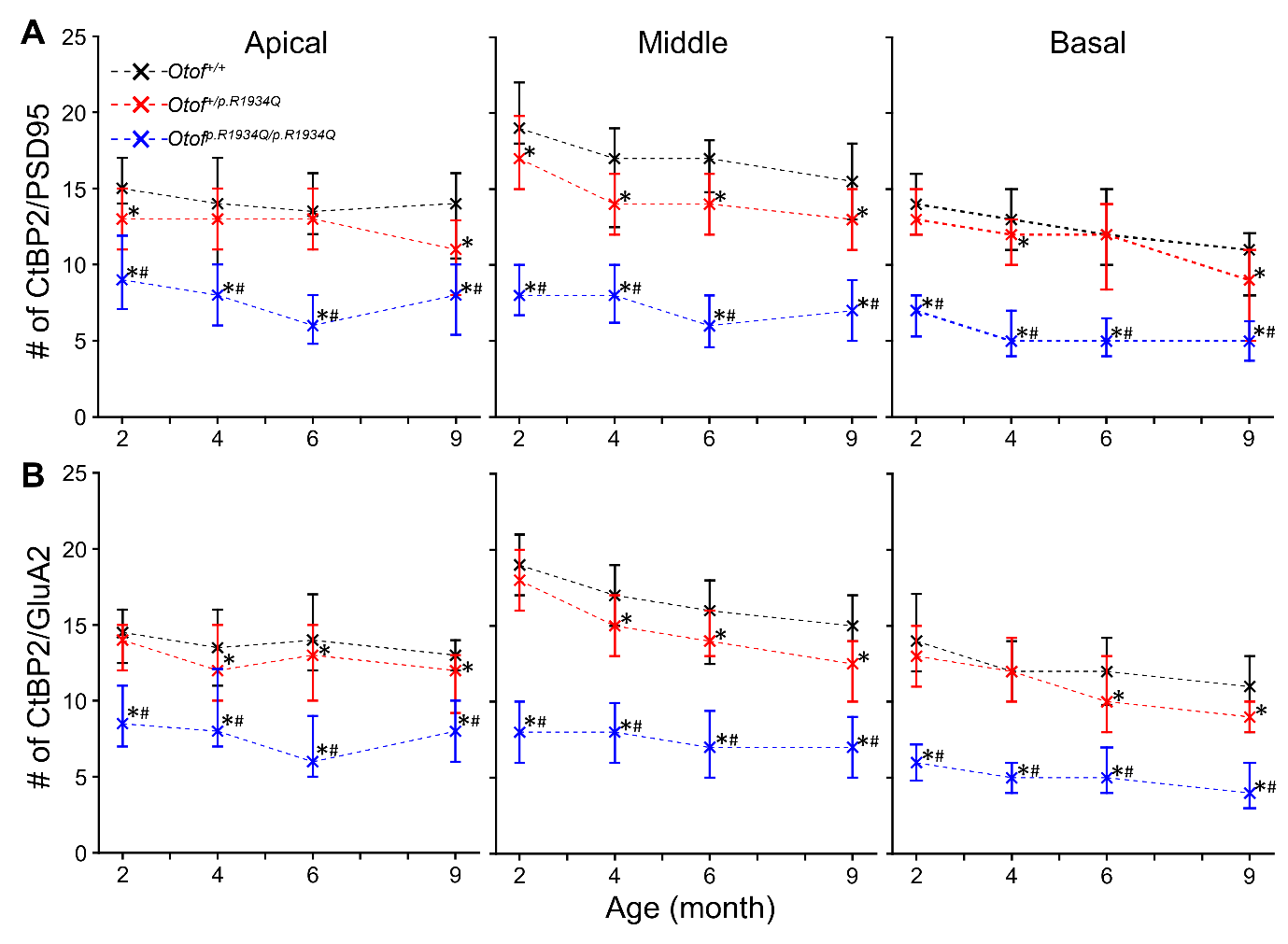
**

**Supplementary figure 4.** Quantification of IHC ribbon synapses of *Otof^+/+^,* *Otof^+/p.R1934Q^*, and *Otof^p.R1934Q/p.R1934Q^.* **A.** The number of paired CtBP2 and PSD95 puncta per IHC. Number of IHCs and cochleae used in the quantification are as following: 2-months-old cochleae (*Otof^+/+^*, 41-57 IHC from n=4; *Otof^+/p.R1934Q^*, 36-54 IHC from n= 4-5; *Otof^p.R1934Q/p.R1934Q^*, 30-42 IHC from n= 5), 4-month-old cochleae (*Otof^+/+^*, 27-34 IHC from n=4; *Otof^+/p.R1934Q^*, 40-59 IHC from n=4-5; *Otof^p.R1934Q/p.R1934Q^*, 31-50 IHC from n=5), 6-month-old cochleae (*Otof^+/+^*, 23-42 IHC from n=4-5; *Otof^+/p.R1934Q^*, 43-60 IHC from n=5-6; *Otof^p.R1934Q/p.R1934Q^*, 34-67 IHC from n=4-6), 9-month-old cochleae (*Otof^+/+^*, 38-63 IHC from n=4-5; *Otof^+/p.R1934Q^*, 23-49 IHC from n=3-6; *Otof^p.R1934Q/p.R1934Q^*, 16-23 IHC from n=3-4). **B.** The number of paired CtBP2 and GluA2 puncta per IHC. Number of IHCs and cochleae used in the quantification are as following: 2 month (*Otof^+/+^*, 38-68 IHC from n=5-6; *Otof^+/p.R1934Q^*, 47-66 IHC from n=5; *Otof^p.R1934Q/p.R1934Q^*, 37-56 IHC from n=4-7), 4-month-old cochleae (*Otof^+/+^*, 60-73 IHC from n=5; *Otof^+/p.R1934Q^*, 52-77 IHC from n=5-6; *Otof^p.R1934Q/p.R1934Q^*, 31-50 IHC from n=5), 6 month apical (*Otof^+/+^*, 47-58 IHC from n=4-5; *Otof^+/p.R1934Q^*, 53-69 IHC from n=6-7; *Otof^p.R1934Q/p.R1934Q^*, 29-58 IHC from n=5), 9-months-old cochleae (*Otof^+/+^*, 38-49 IHC from n=4; *Otof^+/p.R1934Q^*, 29-42 IHC from n=4; *Otof^p.R1934Q/p.R1934Q^*, 18-25 IHC from n=3-4). **A-B.** Data are presented as median (x) and 10-90 percentile range (whiskers). *Otof^+/+^,* black; *Otof^+/p.R1934Q^,* red*; Otof^p.R1934Q/p.R1934Q^,* blue*.* * *p*<0.05 compared to *Otof^+/+^* and # *p*<0.05 compared to *Otof^+/p.R1934Q^*. (ANOVA on Ranks followed by Dunn’s test.)

**
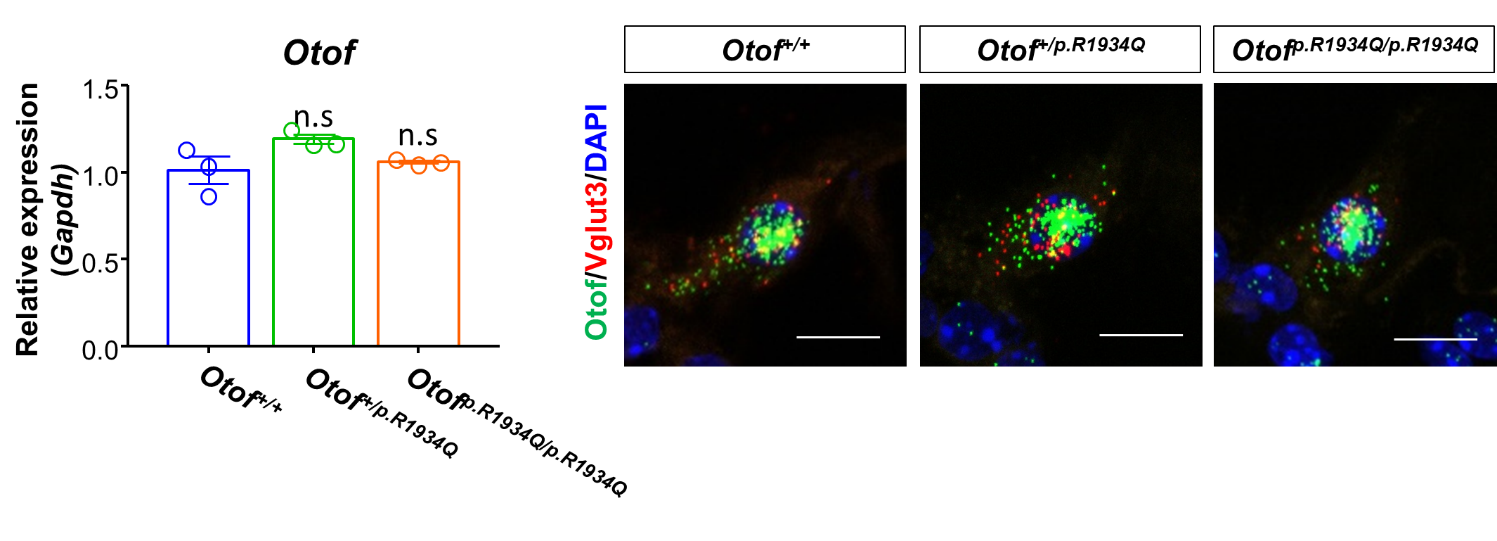
Supplementary Figure 5.** *Otof* mRNA levels quantified with real-time PCR showed no significant differences across genotypes (left, n = 3 replicates, with 4 cochleae pooled per replicate), (one-way ANOVA followed by Tukey’s test) and were visualized using RNAscope (right). Otof (green). Vglut3 (red), DAPI (blue). Scale bar: 20 μm.

**References**

1. Jang MW, Oh DY, Yi E, et al. A nonsense TMEM43 variant leads to disruption of connexin-linked function and autosomal dominant auditory neuropathy spectrum disorder. Proc Natl Acad Sci U S A. 2021;118(22):e2019681118. <https://doi.org/10.1073/pnas.2019681118>

2. Kim BJ, Kim YH, Han JH, et al. Outcome of cochlear implantation in NLRP3-related autoinflammatory inner ear disorders. Otol Neurotol. 2021;42(2):e168-e171. <https://doi.org/10.1097/MAO.0000000000002933>

3. Gavet O, Pines J. Progressive activation of CyclinB1-Cdk1 coordinates entry to mitosis. Dev Cell. 2010;18(4):533-543. <https://doi.org/10.1016/j.devcel.2010.02.013>

4. Ansari N, Müller S, Stelzer EHK, Pampaloni F. Quantitative 3D cell-based assay performed with cellular spheroids and fluorescence microscopy. In: Conn PM, ed. Methods in Cell Biology. Vol 113. Academic Press; 2013:295-309. <https://doi.org/10.1016/B978-0-12-407239-8.00013-6>

5. Akil O, Lustig LR. Mouse cochlear whole mount immunofluorescence. Bio Protoc. 2013;3(5):e332. <https://doi.org/10.21769/bioprotoc.332>

6. Akil O, Chang J, Hiel H, et al. Progressive deafness and altered cochlear innervation in knock-out mice lacking prosaposin. J Neurosci. 2006;26(50):13076-13088. <https://doi.org/10.1523/JNEUROSCI.3746-06.2006>
